# Novel Method in Conferring Degrees

**Published:** 1904-02

**Authors:** 


### NOVEL METHOD IN CONFERRING DEGREES.

The traffic in diplomas has evidently started in a new section of country, if the following quotations indicate that which is implied by the text. It has been rumored for some time that a college in Toronto, Dominion of Canada, would accept legally registered practitioners from any part of the British Colonial possessions and upon an examination confer the degree of Doctor of Dental Surgery (D.D.S.). This, taken at its face value, would seem a harmless proceeding, but when analyzed it assumes special importance and worthy the attention of all interested in keeping the degree in dentistry fully up to the standard established by the National Association of Dental Faculties.

The following is taken from the published announcement of the "Royal College of Dental Surgeons of Ontario:"

"Students of Dentistry *not desiring to qualify for practice in the province of Ontario,* by obtaining the L.D.S. of the R.C.D.S. of Ontario, *may avail themselves of all the privileges of the school of dentistry of the R.C.D.S.* and proceed to the degree of Doctor of Dental Surgery, either in the University of Toronto, or in Trinity University, on compliance with the requirements of their curriculum in dentistry."

The requirement in the announcement of Trinity University, Toronto, is as follows: "All legally qualified practitioners of Dental Surgery of Great Britain, or of any portion of the British empire, will be admitted to the final examination without passing further matriculation and without *further attendance on lectures.* Fee for registration, $5. Fee for final examination, $10. Fee for degree of D.D.S., $15." (Italics ours.)

In a private letter from a prominent official, in another school, to an applicant, the writer warmly endorses this university and its methods.

One of the singular conditions demanded in conferring this degree is to be found in the fact that the recipient is not to make use of it in the Dominion of Canada, but it is available only in other portions of the colonial possessions of Great Britain; in other words, it is not quite good enough for Canada, but will answer very well for outlying districts. This appears to be a remarkable assumption of authority, especially as there is no indication that the people in said sections of the empire have been consulted in the matter. This, however, can be settled by the colonial residents without interference from this side of the boundary line.

It is not supposed that the active originators of this novel method of conferring a degree thought for a moment that they might be imitating the dealers in fraudulent diplomas, but their action comes perilously near to the standard set up by these individuals.

The diplomas sent out from Chicago were not dealt out to persons in the United States, but were considered most suited to the residents of foreign climes. In justice to the schools in Toronto it must be stated that they do require a certain amount of attendance sufficiently long to make an examination. This was not regarded as important by the Chicago diploma manufacturers.

In some of the colonial possessions of Great Britain dentists are trained solely in private practice, and after a certain time are permitted to come up for examination, attendance at a dental college not being required. This is true of New Zealand, where a three years' private preceptorship is demanded, and nothing further except the government examination.

With these facts known to all dental educators, how is it possible for men having a supposed interest in higher dental education to continue such objectionable methods? Whether this plan will tend to attract many from Australia, New Zealand, or South Africa is not important to those dwelling this side of the Dominion, but it is important to these that the degree which originated here be kept unsullied and free from the taint of commercialism. From its adoption as a test of ability, the effort has been to raise its value, and the degree of Doctor of Dental Surgery (D.D.S.) received its highest valuation when a four years' course was demanded of the undergraduates. That the schools in the Dominion of Canada should offer an easy path to its acquirement is not only discreditable, but is in direct violation of the ruling of the National Association of Dental Faculties. To preserve this degree in its purity has been the aim of this organization from its origin, and it cannot view with complacency this open attempt to lessen its value through methods that must be condemned by all dental educators throughout the world.

